# Pulmonary activation of vitamin D_3_ and preventive effect against interstitial pneumonia

**DOI:** 10.3164/jcbn.19-48

**Published:** 2019-09-11

**Authors:** Ichiro Tsujino, Ryoko Ushikoshi-Nakayama, Tomoe Yamazaki, Naoyuki Matsumoto, Ichiro Saito

**Affiliations:** 1 Division of Respiratory Medicine, Department of Internal Medicine, Nihon University School of Medicine, 30-1 Oyaguchi-Kamimachi, Itabashi-ku, Tokyo 173-8610, Japan; 2 Department of Pathology, Tsurumi University School of Dental Medicine, 2-1-3 Tsurumi, Tsurumi-ku, Yokohama, Kanagawa 230-8501, Japan

**Keywords:** vitamin D, interstitial pneumonia, pulmonary fibrosis, prevention

## Abstract

Calcitriol [1,25(OH)_2_D_3_] is usually investigated in studies on the preventive effect of activated vitamin D against interstitial pneumonia. Although cholecalciferol (vitamin D_3_) can be easily obtained in the diet and has a longer half-life than calcitriol, there have been few investigations of its effect on interstitial pneumonia. We used human pulmonary fibroblast cell lines (HPFCs) and a mouse model of bleomycin-induced pulmonary fibrosis to evaluate whether vitamin D_3_ was activated in the lungs and had a preventive effect against interstitial pneumonia. Expression of the vitamin D receptor gene and genes for enzymes metabolizing vitamin D was evaluated in two HPFCs, and the suppressive effect of vitamin D_3_ on induction of inflammatory cytokines was also assessed. Gene expression of the vitamin D receptor and vitamin D-metabolizing enzymes was observed in both human pulmonary fibroblast cell lines. Vitamin D_3_ suppressed bleomycin-induced expression of inflammatory cytokines and fibrosis markers by the HPFCs. In mice, symptoms of bleomycin-induced pulmonary fibrosis were improved and expression of fibrosis markers/fibrosis inducers was decreased by a high vitamin D_3_ diet. Vitamin D_3_ is activated locally in lung tissues, suggesting that high dietary intake of vitamin D_3_ may have a preventive effect against interstitial pneumonia.

## Introduction

In interstitial pneumonia, the alveolar walls become thickened and fibrotic due to inflammation, presumably due to activation of fibroblasts that produce α-smooth muscle actin (αSMA) and type I collagen.^(1)^ Interstitial pneumonia is a complication of Sjӧgren syndrome and the blood level of vitamin D is lower in Sjӧgren patients than in healthy people,^(2)^ so it has been suggested that interstitial pneumonia may be associated with vitamin D deficiency.^(3)^

Vitamin D is a fat-soluble vitamin that facilitates absorption of calcium and phosphorus from the intestinal tract, promotes parathyroid hormone production and secretion, and activates bone remodeling by osteoblasts.^(4)^ It was recently reported that vitamin D deficiency not only affects bone metabolism, but also has a role in lifestyle-related diseases, including cardiac disease, diabetes, and cancer, and that vitamin D can prevent infections.^(5,6)^

The physiologically active form of vitamin D is calcitriol [1,25(OH)_2_D_3_], which is used to treat osteoporosis. However, the half-life of calcitriol is only 15 h and maintaining a stable blood level is difficult, so the expected efficacy may not be achieved.^(7)^ Cholecalciferol (VD_3_) is produced in the skin after exposure to sunlight or can be taken orally and undergoes hepatic metabolism/isomerization to become calcidiol [25(OH)VD_3_], a circulating form of vitamin D with a long half-life of 15 days. Then 25(OH)VD_3_ is activated locally in various tissues to show its physiological effects.^(8)^

Pulmonary injury caused by inhalation of lipopolysaccharide is more severe in mice with vitamin D receptor (VDR) knockout than in wild-type mice,^(9)^ and the severity of such pulmonary injury in mice is correlated with the blood level of 25(OH)VD_3_.^(10)^ Previous studies on pulmonary fibrosis have evaluated the anti-inflammatory and anti-fibrotic effects of 1,25(OH)_2_D_3_ on pulmonary fibroblasts,^(11)^ and the preventive effect of 1,25(OH)_2_D_3_ administered by oral gavage in a mouse model of bleomycin-induced pulmonary fibrosis.^(12)^ However, few studies have assessed the influence of high dietary intake of VD_3_ on pulmonary fibrosis.

Therefore, we conducted an *in vitro* study using human pulmonary fibroblast cell lines and also employed mice with bleomycin-induced pulmonary fibrosis to evaluate whether dietary VD_3_ was metabolized to active 1,25(OH)_2_D_3_ in the lungs and whether it prevented interstitial pneumonia.

## Materials and Methods

### *In vitro* study

#### Cell culture

A normal human fetal pulmonary fibroblast cell line (MRC-5) and an immortalized cell line derived from MRC-5 (MRC-5 SV1 TG1) (KAC Co., Ltd., Kyoto, Japan) were maintained in α-minimal essential medium (α-MEM) containing 10% fetal calf serum (FCS) and antibiotics (Life Technologies Japan Ltd., Tokyo, Japan) at 37°C under 5% CO_2_. MRC-5 SV1 TG1 cells were suspended in α-MEM containing 1% charcoal/dextran-treated FBS (HyClone Laboratories, Inc., South Logan, UT), and the suspension was seeded into a 6-well plate at 2 × 10^5^/well. Then incubation was done for 48 h after 25 µg/ml bleomycin (Nippon Kayaku, Co., Ltd., Tokyo, Japan) was added together with 50 ng/ml cholecalciferol (VD_3_) (Merck KGaA, Darmstadt, Germany), 50 pg/ml calcitriol [1,25(OH)_2_D_3_] (Merck KGaA, Darmstadt, Germany) or the vehicle only. In an independent experiment, bleomycin was added to cells that had been pretreated overnight with cholecalciferol, calcitriol, or the vehicle, and the cells were subsequently cultured for 24 h.

#### Analysis of gene expression in human pulmonary fibroblasts

RNA harvested from MRC-5 cells or MRC-5 SV1 TG1 cells was purified and cDNA was synthesized from each RNA sample (1 µg) by using a SuperScript VILO cDNA Synthesis Kit (Life Technologies Japan Ltd., Tokyo, Japan). Using the cDNA as a template, RT-PCR was performed with Ex-Taq DNA polymerase (Takara Bio Inc., Shiga, Japan) to assess expression of mRNA for the VDR and vitamin D-metabolizing enzymes (CYP27A1, CYP2R1, and CYP27B1).

cDNA was also synthesized from RNA that had been harvested from MRC-5 SV1 TG1 cells treated with bleomycin, and RT-PCR was performed to investigate the expression of genes for various molecules related to fibrosis and inflammation [α smooth muscle actin (αSMA), type I collagen alpha 2 chain (COL1A2) , secreted phosphoprotein 1 (SPP1), interleukin-1β (IL-1β), transforming growth factor-β1 (TGF-β1), and β-actin]. The primers for RT-PCR are shown in Table 1.

PCR products were run on agarose gel containing 0.01% Gel-Red (Biotium Inc., Hayward, CA) and UV images were obtained with a gel documentation system. Band intensities of the PCR products were converted to numerical data by using Image Studio software (LI-COR, Inc., Lincoln, NE), and gene expression levels were compared between the groups.

### *In vivo* study

#### Animals

Five-week-old male C57BL/6JJcl mice (*n* = 15) (CLEA Japan, Inc., Tokyo, Japan) were housed under specific pathogen free conditions at a temperature of 23 ± 2°C and a humidity of 60 ± 15% with a 12-h light-dark cycle, and were allowed free access to water and a special AIN-93G diet containing VD_3_ (200 IU/100 g).

#### High vitamin D diet and induction of pulmonary fibrosis by bleomycin

**Fig. 1:**
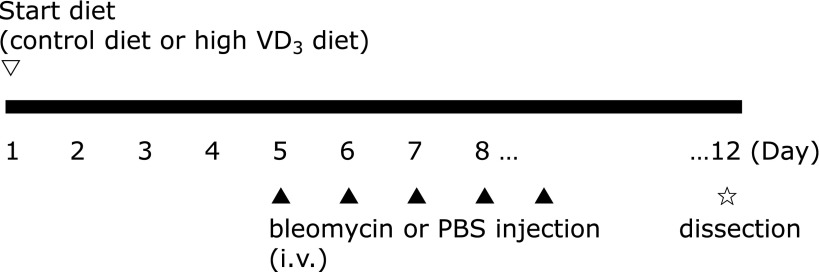
Schedule of tests in the mouse model of bleomycin-induced pulmonary fibrosis. Six-week-old C57BL/6JJcl mice were randomized to three groups (*n* = 5 each): a control (CTL) group (control diet + PBS i.v.), a bleomycin (BLM) group (control diet + bleomycin i.v.), and a high VD_3_ + bleomycin (VD + BLM) group (high VD_3_ diet + bleomycin i.v.). The control diet contained 200 IU/100 g of VD_3_ and the high VD_3_ diet contained 1,000 IU/100 g. From four days after starting each diet, bleomycin (10 mg/kg) was administered via the tail vein once daily for five days in the BLM and VD + BLM groups, while PBS was administered in the CTL group. Lungs were harvested at four days after the finish of administration.

At 6 weeks old, mice were assigned to the following three groups (*n* = 5 each): a group treated with bleomycin and given a high VD_3_ diet (high VD_3_ + bleomycin group), a group treated with bleomycin that remained on the basal diet (bleomycin group), and an untreated control group that remained on the basal diet (control group). In the high VD_3_ + bleomycin group, the mice received a diet with 1,000 IU/100 g of VD_3_ vs 200 IU/100 g in the other groups. From Day 4 on the assigned diet, bleomycin (10 mg/kg) was injected into the tail vein once daily for five days in the high VD_3_ + bleomycin group and the bleomycin group, while PBS was administered to the control group. At four days after finishing administration, the mice were sacrificed humanely under deep sevoflurane anesthesia and the lungs were harvested (Fig. 1).

All applicable international, national, and/or institutional guidelines for the care and use of animals were followed. All procedures performed in studies involving animals were in accordance with the ethical standards of the institution or practice at which the studies were conducted. The animal experiment was approved by the committee for animal experiments at Tsurumi University (Permission number: 29A043).

#### Histopathological analysis of fibrosis

Mouse lungs were fixed in 4% paraformaldehyde in PBS, cut into two pieces (coronal section), and embedded in paraffin. Then the blocks were cut into 4 µm sections that were stained with hematoxylin and eosin (HE) for light microscopy, and digital photographs were taken under a 20× objective. The photographs were analyzed with Image J software,^(13)^ and alveolar wall thickening was assessed from the percent area of HE-stained tissue in the overall field. Other sections were stained with Sirius red/fast green stain (Sirius Red/Fast Green Collagen Staining Kit, Condrex, Inc., Redmond, WA), and the severity of pulmonary fibrosis (including the collagen content) was evaluated with the modified Ashcroft scale according to the method of Hübner *et al.*^(14)^ Briefly, sections were observed at 200× magnification and the extent of pulmonary fibrosis in each field was scored from 0 (normal) to 8 (complete fibrosis), after which the mean score was calculated from the scores for all sections of each specimen. The person performing histological evaluation was blinded to information about the specimens.

#### Analysis of gene expression in mouse lung tissue

Total RNA was extracted from mouse lung tissues, and cDNA was synthesized as mentioned above. Then RT-PCR was performed using this cDNA and the primers shown in Table 1, after which the levels of TGF-β1, SPP1, IL1-β, and β-actin mRNA expression were determined. Band intensities of the PCR products on agarose gels were converted to numerical data by using Image Studio software, and gene expression was compared between the groups.

#### Statistical analysis

In the *in vivo* study, mean values of parameters were compared between the groups by the *t* test or Mann-Whitney *U* test, as appropriate, and the extent of changes was evaluated by calculation of Cohen’s *d*.

## Results

### Expression of the vitamin D receptor and vitamin D-metabolizing enzymes

**Fig. 2:**
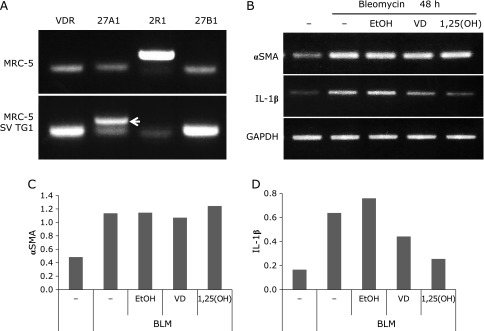
Expression of the vitamin D receptor and vitamin D-metabolizing enzymes in human fibroblasts, and VD_3_-dependent suppression of the induction of IL-1β gene expression in pulmonary fibroblasts by bleomycin. (A) To assess gene expression in MRC-5 and MRC-5 SV1 TG1 cells, RT-PCR was performed using specific primers for the vitamin D receptor (VDR) or vitamin D-metabolizing enzymes, including CYP27A1 (27A1), CYP2R1 (2R1), and CYP27B1 (27B1). (B) MRC-5 SV1 TG1 cells were treated with 25 µg/ml bleomycin for 48 h and RT-PCR was performed using specific primers for αSMA, IL-1β, and GAPDH. (C, D) Band intensities of the PCR products were converted to numerical data by image analysis software, and the data for αSMA and IL-1β were normalized by the GAPDH value.

In MRC-5 cells, VDR expression was detected, as well as expression of CYP2R1 [an enzyme metabolizing VD_3_ to 25(OH)D_3_] and CYP27B1 [an enzyme metabolizing 25(OH)D_3_ to 1,25(OH)_2_D_3_]. MRC-5 SV1 TG1 cells showed expression of the vitamin D receptor and vitamin D-metabolizing enzymes CYP27A1and CYP27B1, but not CYP2R1 (Fig. 2A).

### Effect of VD_3_ in an *in vitro* model of pulmonary fibrosis

After 48 h of treatment with bleomycin, MRC-5 SV1 TG1 cells showed elevated expression of IL-1β (an inflammatory cytokine) and αSMA (a marker of myofibroblast differentiation), while treatment with VD_3_ or 1,25(OH)_2_D_3_ suppressed IL-1β expression (Fig. 2B–D).

**Fig. 3:**
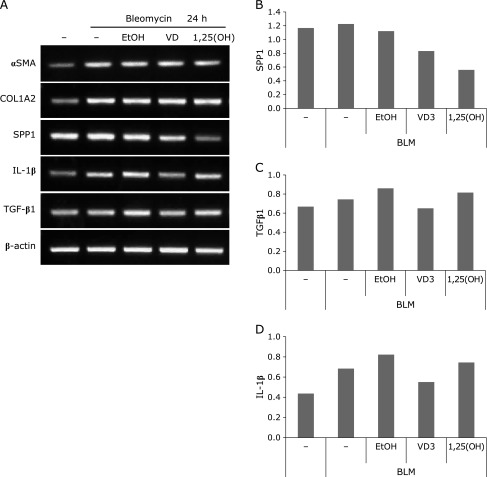
Pretreatment of pulmonary fibroblasts with VD_3_ suppresses bleomycin-induced expression of pro-fibrotic genes, fibrosis markers, and inflammatory markers. (A) MRC-5 SV1 TG1 cells underwent pretreatment with VD_3_ or 1,25(OH)_2_D_3_ overnight and then were incubated with 25 µg/ml bleomycin for 24 h, after which RT-PCR was performed using specific primers for αSMA, COLA2, SPP1, IL-1β, TGF-β1, and β-actin (ACTB). (B–D) Band intensities of the PCR products were converted to numerical data by image analysis software, and the data for SPP1, IL-1β, and TGF-β1 were normalized by the β-actin value.

Pretreatment of cells with VD_3_ or 1,25(OH)_2_D_3_ did not suppress expression of αSMA or COL1A2 (an extracellular matrix protein that increases with fibrosis). However, pretreatment suppressed the expression of SPP1 (another extracellular matrix protein) and IL-1β (Fig. 3).

### Histopathological analysis of pulmonary fibrosis

**Fig. 4:**
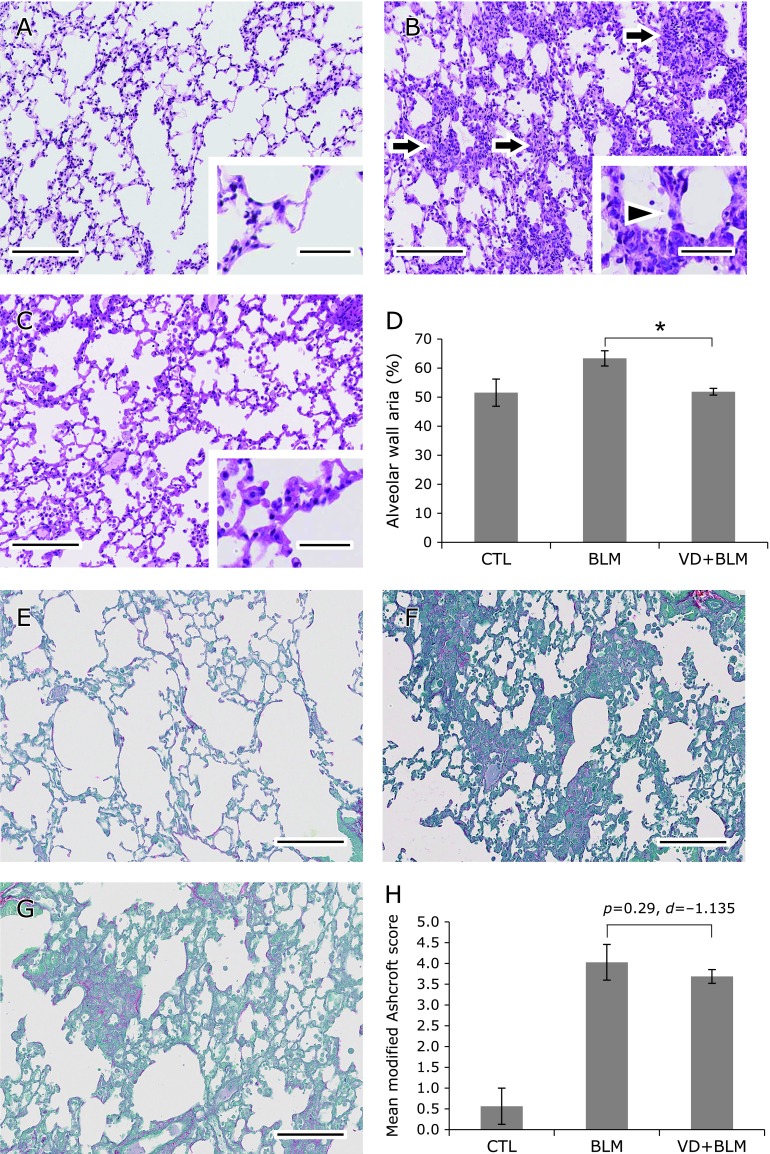
High VD_3_ diet suppresses alveolar wall thickening in mice with bleomycin-induced pulmonary fibrosis and improves the fibrosis scores estimated by the modified Ashcroft method. (A–C) Representative specimens of lung tissue from each group with HE staining (bar = 100 µm; bar in the inset = 30 µm). (A) Control group. (B) Bleomycin group. (C) High VD_3_ + bleomycin group. (A) Obvious lesions were not observed in the lungs of the control group. (B) Alveolar wall thickening (arrowhead) and multiple fibrotic lesions (arrows) were observed in the bleomycin group. (C) Histological changes were milder in the high VD_3_ + bleomycin group compared with the bleomycin group. (D) Image analysis (Image J) of the HE-stained area of alveolar walls in the microscopic field shown as a percentage. The value showing maximum deviation from the mean was excluded in each group. CTL: control group; BLM: bleomycin group; VD + BLM: high VD_3_ + bleomycin group. The alveolar wall area was 51.5 ± 4.7% in the control group, 63.4 ± 2.6% in the bleomycin group, and 51.9 ± 1.2% in the high VD_3_ + bleomycin group. *p = 0.034 (Mann-Whitney *U* test), and Cohen’s *d* = –6.108. (E–G) Representative specimens of lung tissues from each group with Sirius red/fast green staining (bar = 100 µm). (E) Control group (score = 1). (F) Bleomycin group (score = 5). (G) High VD_3_ + bleomycin group (score = 4). Compared with the control group, the alveolar walls are thicker and there are more fibrotic lesions in the other groups. (H) Comparison of the mean modified Ashcroft score. The value showing maximum deviation from the mean was excluded in each group. The mean score was 0.716 ± 0.583 in the control (CTL) group, 3.818 ± 0.474 in the bleomycin (BLM) group, and 3.794 ± 0.231 in the high VD_3_ + bleomycin (VD + BLM) group.

 Compared with the control group, the bleomycin group showed alveolar wall thickening and narrowing of alveolar spaces due to fibrosis in HE-stained sections, as well as an increase of Type 2 alveolar epithelial cells and fibrotic lesions. These histological changes were suppressed in the high VD_3_ + bleomycin group. When alveolar wall thickening was evaluated by image analysis, the stained area of the alveolar wall was about 12% larger in the bleomycin group (63.4 ± 2.6%) than in the control group (51.5 ± 4.7%), but was significantly smaller in the high VD_3_ + bleomycin group (51.9 ± 1.2%) than the bleomycin group (*p* = 0.034, *d* = –6.108) (Fig. 4A–D).

When pulmonary fibrosis was evaluated using the modified Ashcroft scale, the mean score was 0.716 ± 0.583 in the control group vs 3.818 ± 0.474 in the bleomycin group and 3.794 ± 0.231 in the high VD_3_ + bleomycin group. While both groups showed progression of fibrosis, the mean score was lower in the high VD_3_ + bleomycin group (*p* = 0.29, *d* = –1.135) (Fig. 4E–H).

### Effect of a high VD_3_ diet on gene expression

**Fig. 5:**
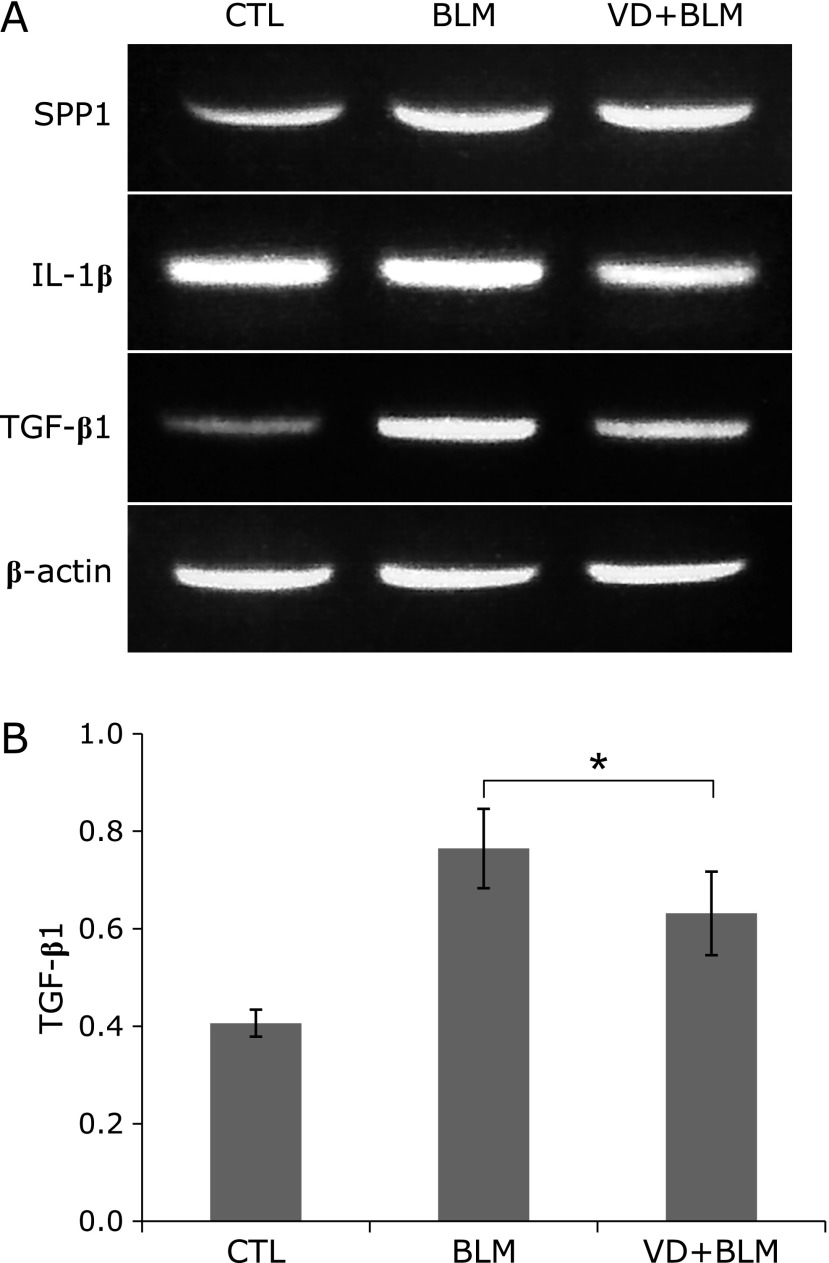
High VD_3_ diet suppresses expression of TGF-β1 in mice with bleomycin-induced pulmonary fibrosis. Representative result of RT-PCR performed using lung tissues from mice in each group. CTL: control group; BLM: bleomycin group; VD + BLM: high VD_3_ + bleomycin group. (A) RT-PCR with specific primers for SPP1, IL-1β, and TGFβ1. (B) Band intensities of the PCR products were converted to numerical data by image analysis software and each value was normalized by the β-actin value. Outliers were excluded before the data were compared. *p = 0.025 (*t* test), Cohen’s *d* = –1.32.

 Expression of mRNA for SPP1 (a fibrosis marker) was increased in the bleomycin group and in the high VD_3_ + bleomycin group. There was no difference of IL-1β mRNA expression among the three groups. TGF-β1mRNA expression was increased in the bleomycin group compared with the control group, but was significantly lower in the high VD_3_ + bleomycin group than in the bleomycin group (*p = 0.025, *d* = –1.32) (Fig. 5).

## Discussion

In this study, expression of VDR and enzymes involved in vitamin D activation was evaluated in MRC-5 cells (normal human lung-derived fibroblasts) and MRC-5 SV1 TG1 cells (an immortalized cell line derived from MRC-5). Both cell lines showed expression of VDR and CYP27A1 or CYP2R1 [enzymes metabolizing VD_3_ to 25(OH)D_3_], and expression of CYP27B1 [an enzyme metabolizing 25(OH)D_3_ to 1,25(OH)_2_D_3_] was also observed (Fig. 1). These findings suggested that pulmonary fibroblasts could metabolize VD_3_ to active vitamin D.

Expression of the inflammatory marker IL-1β was induced in MRC-5 SV1 TG1 cells by bleomycin, but was suppressed by 1,25(OH)_2_D_3_, consistent with a previous report,^(11)^ and IL-1β expression was also suppressed by VD_3_ (Fig. 2). These findings suggested that VD_3_ could be metabolized to 25(OH)D_3_ and locally transformed to 1,25(OH)_2_D_3_ by pulmonary fibroblasts, thus acquiring bioactivity in the lungs without requiring metabolism by the kidney.

To assess the preventive effect of vitamin D, MRC-5 SV1 TG1 cells were pretreated with VD_3_ or 1,25(OH)_2_D_3_ before exposure to bleomycin. VD_3_ pretreatment suppressed bleomycin-induced expression of IL-1β, but did not suppress expression of αSMA (a marker of fibroblast activation) or collagens. However, VD_3_ suppressed expression of SPP1, the gene for osteopontin, an extracellular matrix protein that increases at sites of advanced fibrosis and promotes migration of activated fibroblasts to fibrotic foci.^(15)^ Our findings suggested that VD_3_ prophylaxis may not suppress bleomycin-induced activation of pulmonary fibroblasts, but can inhibit inflammation and accumulation of activated fibroblasts.

In a previous *in vivo* study, 1,25(OH)_2_D_3_ was administered to mice with bleomycin-induced pulmonary fibrosis, and immunostaining of lung tissues showed a decrease of αSMA-positive cells probably because suppression of osteopontin expression by 1,25(OH)_2_D_3_ reduced the accumulation of activated fibroblasts.^(12)^ The *in vitro* experiment performed in this study showed little suppression of inflammatory markers by 1,25(OH)_2_D_3_ administration, probably due to the influence of the time lag from addition of 1,25(OH)_2_D_3_ to addition of bleomycin (overnight) and the short half-life of 1,25(OH)_2_D_3_.

Therefore, VD_3_ with its longer half-life could be more suitable for prophylaxis than 1,25(OH)_2_D_3_, provided that target tissues possess enzymes to activate VD_3_.

This study suggested that VD_3_ could be metabolized to 25(OH)VD_3_ (calcidiol) and to physiologically active 1,25(OH)_2_D_3_ by enzymes expressed in pulmonary fibroblasts, and demonstrated suppression of IL-1 and SPP1 expression in these cells, indicating that VD_3_ may prevent interstitial pneumonia. CYP27B1 is expressed in the prostate gland where it metabolizes 25(OH)D_3_ to 1,25(OH)_2_D_3_, after which 1,25(OH)_2_D_3_ inhibits cell growth and carcinogenesis.^(16)^ Thus, the effects of localized vitamin D activation in various cells or tissues may warrant further investigation.

We also studied the effect of VD_3_ in mice with bleomycin-induced pulmonary fibrosis. Suppression of fibrosis by high oral intake of VD_3_ was confirmed histologically. In addition, RT-PCR showed that a high VD_3_ diet decreased TGF-β1 expression. Zhang *et al.*^(12)^ also reported a suppressive effect of vitamin D on pulmonary fibrosis. However, they administered 1,25(OH)_2_D_3_ by oral gavage, while mice obtained VD_3_ from their diet in our study. There were differences of IL-1β and SPP1 expression from our *in vitro* findings. It is possible that *in vivo* IL-1β and SPP1 expression could have been suppressed by VD_3_ if the dose was higher or if we had studied a larger number of mice or performed sampling at a different time.

It was recently reported that impairment of vitamin D receptor signaling due to reduced receptor expression and a decrease of its ligand causes excessive TGF-β signaling and abnormal activation of fibroblasts in patients with systemic sclerosis.^(17)^ In addition, the active vitamin D analogue maxacalcitol was found to improve renal tubular interstitial fibrosis in the obstructed kidney by recruiting the smad3 phosphatase-VDR complex to pSmad3.^(18)^ On the other hand, chronic dietary vitamin D deficiency led to RAS activation with induction of TGF-β1 expression and activation of a pro-fibrotic cascade has been reported.^(19)^ It was also reported that Wnt/β-catenin signaling is involved in tissue fibrosis independently of smad,^(20,21)^ and that 1,25(OH)_2_D_3_ inhibits Wnt/β-catenin signaling.^(22)^ Further investigation will be required to determine which of these pathways was relevant to the inhibition of fibrosis observed in the present study.

In conclusion, our experiments using cell lines and a mouse model suggested that vitamin D could be activated in the lungs and that dietary intake of vitamin D might prevent interstitial pneumonia by suppressing pulmonary fibrosis. Oral intake of vitamin D may be useful for prevention or treatment of various diseases.

## Figures and Tables

**Table 1:** Sequences of the primers used for gene expression analyses

Species	Target	Forward primer (5'→3')	Reverse primer (5'→3')	Size (bp)
Human	VDR	TCTCCAATCTGGATCTGAGTGAA	GGATGCTGTAACTGACCAGGT	111
	CYP27A1	GGCAAGTACCCAGTACGG	AGCAAATAGCTTCCAAGG	292
	CYP2R1	AGAGACCCAGAAGTGTTCCAT	GTCTTTCAGCACAGATGAGGTA	259
	CYP27B1	GGAACCCTGAACAACGTAGTC	AGTCCGAACTTGTAAAATTCCCC	119
	αSMA	GACCCTGAAGTACCCGATAGAAC	GGGCAACACGAAGCTCATTG	98
	COL1A2	GGCCCTCAAGGTTTCCAAGG	CACCCTGTGGTCCAACAACTC	166
	SPP1	GCCGAGGTGATAGTGTGGTT	TGAGGTGATGTCCTCGTCTG	101
	IL-1β	ATGATGGCTTATTACAGTGGCAA	GTCGGAGATTCGTAGCTGGA	132
	ACTB	ATAGCACAGCCTGGATAGCAACGTAC	CACCTTCTACAATGAGCTGCGTGTG	158
	GAPDH	CCATGGAGAAGGCTGGGG	CCAAAGTTGTCATGGATGACC	196
Human-mouse	TGF-β1	GGCCAGATCCTGTCCAAGC	GTGGGTTTCCACCATTAGCAC	201
Mouse	SPP1	TCACCATTCGGATGAGTCTG	ACTTGTGGCTCTGATGTTCC	437
	IL-1 β	CACAGCAGCACATCAACAAG	GTGCTCATGTCCTCATCCTG	118
	ACTB	TGTTACCAACTGGGACGACA	CTGGGTCATCTTTTCACGGT	139
